# Stevens Johnson Syndrome associated with Lamotrigine

**DOI:** 10.12669/pjms.296.4385

**Published:** 2013

**Authors:** Shama Parveen, M. Afzal Javed

**Affiliations:** 1 Dr. Shama Parveen, The Medical Centre, Manor Court Avenue, Nuneaton, CV11 5HX, UK.; 2 Dr. M. Afzal Javed, The Medical Centre, Manor Court Avenue, Nuneaton, CV11 5HX, UK.

**Keywords:** Stevens-Johnsons syndrome, Hypersensitivity reaction, Lamotrigine

## Abstract

Stevens-Johnsons Syndrome (SJS) is an immune-complex-mediated hypersensitivity reaction and has been linked as an adverse side effects to many drugs. Lamotrigine, an anticonvulsive medication and also a commonly used mood stabiliser, can be associated with this adverse reaction. Although this has not been reported very commonly , SJS has high mortality and morbidity and requires careful attention as the use of Lamotrigine is increasing in clinical practice. We present a case where the patient developed Stevens - Johnson Syndrome three weeks after being started on Lamotrigine. The case is discussed for its relevance to the use of Lamotrigine which is currently prescribed very commonly in psychiatric practices.

## INTRODUCTION

Stevens–Johnson Syndrome (SJS), a dermatological emergency is a rare condition; with a reported incidence of around 2.6 to 6.1 cases per million people per year with a mortality rate of around 5%.^1^ SJS is named after two American pediatricians, Albert Mason Stevens and Frank Chambliss Johnson, who jointly published the first description of the disorder in the American Journal of Diseases of Children in 1922.^2^ Stevens-Johnson Syndrome (SJS) is an immune-complex-mediated hypersensitivity reaction that characteristically involves skin and mucous membrane. The main known cause is hypersensitivity to certain drugs, followed by infections and, rarely, cancers.^3^^,^^4^

Although reported as a rare event Stevens–Johnson Syndrome (SJS) has been found to be more common in adults than in children. Women are affected more often than men, with cases occurring at a two to one (2:1) ratio. SJS is linked to a number of drugs, infections & carcinomas; people with AIDS are also at an increased risk of developing this disorder.^1^

SJS and toxic epidermal necrolysis (TEN) are two forms of this life-threatening skin condition, in which cell death causes the epidermis to separate from the dermis. Toxic Epidermal Necrolysis (TEN) is a more severe form of SJS and may be associated with high morbidity and mortality. Toxic Epidermal Necrolysis (TEN) can be classified by the extent of body surface area detachment as SJS - A "minor form of TEN," with less than 10% body surface area (BSA) detachment, Overlapping SJS/TEN - Detachment of 10-30% BSA and TEN - Detachment of more than 30% BSA.^5^

Clinical features of SJS include significant involvement of skin and oral, nasal, eye, vaginal, urethral, GI, and lower respiratory tract mucous membranes. Lesions may continue to erupt in crops for as long as 2-3 weeks. Skin rash can begin as macules that develop into papules, vesicles, bullae, urticarial plaques or confluent erythema. Bullous lesion can rupture and may lead to further complications. Mucosal involvement includes erythema, oedema, sloughing, blistering, ulceration and necrosis. GI and respiratory involvement may progress to necrosis. SJS is a serious systemic disorder with the potential for severe morbidity and even death. Whereas Toxic Epidermal Necrolysis has a higher mortality (30-35%); Stevens-Johnson Syndrome and transitional forms correspond to the same syndrome, but with less extensive skin detachment and a lower mortality (5-15%).^6^

SJS has been commonly reported as an adverse reaction to a number of drugs, and carcinomas & infections have also been linked to its occurrence. Drug aetiologies include reaction to drugs including Penicillin, Sulphonamide, Phenytoin, Valproate, Carbamazepine, non-steroidal anti-inflammatory drugs, anti-malarial and allopurinol.^7^^,^^8^ Coxsackievirus, Echovirus, Herpes Simplex viruses and Mycoplasma infections have also been linked to this syndrome in some cases. Similarly SJS has also been associated with immunisation, e.g. measles and hepatitis B.^9^ However, in approximately 25 to 50 percent of cases no cause can be identified.

Anti-epileptics as a group have been shown to cause SJS with variation in its association with individual anti epileptic medications.^8^ Although present data supports an association of SJS with combined use of all antiepileptic drugs, the data for individual drugs are not convincing because of small numbers of exposed patients and an inability to adjust for possible confounding variables.^10^ Among antiepileptic drugs, SJS has been generally reported with the use of Carbamazepine, Phenobarbital and Phenytoin especially during the early period or start of these medications (usually the first few days or few weeks). Lamotrigine, another commonly used antiepileptic drug and also an emerging treatment for Depression has not been well reported to be associated with SJS in medical literature although there have been some reports linking Lamotrigine with this syndrome. We are presenting a case where a patient developed SJS after 3-4 weeks of use of Lamotrigine. Although she showed significant improvement in her mental state after the addition of Lamotrigine, her initial response during the first 2-3 weeks did not show any signs of this syndrome.

## CASE REPORT

This 56 years old lady who has been known to the local mental health services for the past 20 years with symptoms of depression and anxiety was attending follow up appointments on and off. For her current episode she was in contact with the local team for the last few years and had presented with moderate to severe depression that needed regular follow up and review of her medication. She also had two inpatient hospital admissions to the psychiatric unit after attempting suicide on two occasions during this episode. During her latest admission, she was treated with different antidepressants and anxiolytics but without any major benefit. Because of the poor control of symptoms of depression, Lamotrigine was added to her other medications (Sertaline and Buspirone which she had been taking for many months). She was started on lamotrigine 25 mg daily which was gradually increased to 50 mg twice daily over the next 2 weeks. Her anxiety and depressive symptoms showed significant improvement on this combination and she started feeling almost back to her normal self. She was continued on this treatment & was discharged from the psychiatric inpatient unit on a combination of Lamotrigine 50mg twice a day, Sertaline 150 mg daily and Buspirone 5mg twice daily. At her weekly follow up, she continued showing improvement in her mental state and the treating team was very satisfied with her response to treatment.

About 16 days following hospital discharge she developed conjunctivitis and over the next 3-4 days, developed swelling of the face and lips. She also developed erosion of the mucous membrane inside her mouth & erythematous papules and bullous eruptions over her body that were particularly bad on the palms of her hands and soles of her feet where she developed detachment of the epidermis. In view of her increasing symptoms, she had to be referred to the general hospital on the 4th day after developing these side effects. She was admitted for further treatment. She had to be treated in the Intensive Care Unit as her physical symptoms deteriorated over the next few days. All her routine blood tests were normal except C-Reactive Protein which was high. She was kept under the care of a medical specialist, ophthalmologist and dermatologist who agreed with the clinical diagnosis of Stevens - Johnson Syndrome. Lamotrigine, along with her other antidepressant medications was stopped. After a few days she started feeling better, made a full recovery in 2 weeks time and was discharged home. Her ophthalmic symptoms needed a few more weeks for full recovery.

## DISCUSSION

Lamotrigine is an anti epileptic medication which is also used as a mood stabilizer.^11^ Side effects of Lamotrigine generally include CNS symptoms like headache, fatigue, dizziness, sleep disturbance, tremor, movement disorder, agitation, confusion, hallucinations, Gastrointestinal symptoms like diarrhoea, nausea, vomiting, hepatic dysfunction and skin & cutaneous side effects like rash. Stevens-Johnson syndrome has also been mentioned as a rare hypersensitivity reaction/ side effect in the drug information pack of Lamotrigine characterised by severe rash, fever, lymphadenopathy, hepatic dysfunction, blood disorder, and Disseminated Intravascular Coagulation with multi organ dysfunction. 

Hypersensitivity reactions can happen with almost all antiepileptic drugs with cutaneous side effects occurring in 3 to 10% patients and rash usually developing in the first few weeks in a small number of patients.^8^^,^^12^ It has also been mentioned that SJS can turn into more severe Toxic Epidermal Necrolysis (TEN).^1^^,^^6^ However not many reports have been published suggesting SJS as a common side effect of the use of Lamotrigene. SJS, on the other hand, has been reported from concomitant use of Valproic Acid and Lamotrigine^13^^,^^14^ and it is thought that Valproic Acid interferes with the metabolism of Lamotrigine by inhibiting glucuronide causing increased Lamotrigine blood levels.^15^^,^^16^ It has also been suggested that rapid dose escalation of Lamotrigine increases the risk of cutaneous rash.^14^^,^^17^^,^^18^

In our case, the patient was on Sertraline and Buspirone for many months and did not show any side effects suggestive of a drug reaction. There is also no evidence in the literature that either Sertraline or Buspirone can cause SJS or concomitant use of these medications with Lamotrigine increases the risk of SJS.

Rapid dose escalation is usually linked to the increased the risk of coetaneous side & dose escalation in our case was done as 25mg once daily for a week, followed by 50mg once daily for a week and then it was increased to 50mg twice daily. All her symptoms developed after about two week of being on Lamotrigine (50 mg twice daily dose).

We are reporting this case, since there is increasing use of Lamotrigine in psychiatry (especially as a mood stabilizer) and because of the rarity of Stevens–Johnson Syndrome (SJS) as an adverse effect of Lamotrigine. Mechanisms for Lamotrigine-induced SJS are less well understood but recent evidence suggests that antiepileptic drug-related hypersensitivity may be a consequence of chemotoxic and immunologically mediated injury; however, the pathogenesis of this reaction may vary somewhat among different antiepileptic drugs.^19^ It is very easy to forget about this important side effect which can have very high mortality and morbidity.

The risk of developing SJS with Lamotrigine is rare and relatively predictable during the first few weeks of its use; clinicians prescribing this medication should however be aware of this high risk condition. Current evidence indicates that although Lamotrigine may cause a serious rash for which clinicians must continue to observe standard new dosing paradigms & practice precautions, in the case of non-serious rash, a re-challenge with Lamotrigine can also be considered^20^ in many cases.
